# Para Kala-Azar Dermal Leishmaniasis: A Case Report

**DOI:** 10.7759/cureus.33701

**Published:** 2023-01-12

**Authors:** Md Moniruzzaman, S.K. Jakaria Been Sayeed, Md. Abdur Rahim, Rashedul Hassan, Md. Mujibur Rahman

**Affiliations:** 1 Internal Medicine, National Institute of Neuro Sciences & Hospital, Dhaka, BGD; 2 Internal Medicine, Shaheed Suhrawardy Medical College and Hospital, Dhaka, BGD; 3 Internal Medicine, Green Life Medical College Hospital, Dhaka, BGD; 4 Internal Medicine, Bangabandhu Sheikh Mujib Medical University Hospital, Dhaka, BGD

**Keywords:** liposomal amphotericin b, leishmania donovani, para kala-azar, post kala-azar dermal leishmaniasis, visceral leishmaniasis

## Abstract

Rarely, post-kala-azar dermal leishmaniasis (PKDL) may coexist with visceral leishmaniasis (VL). The concomitant PKDL and VL are referred to as Para-kala-azar dermal Leishmaniasis. We report a case of Para kala-azar dermal leishmaniasis in a chronic Hepatitis-B virus-infected patient who presented with an abdominal lump and multiple maculopapular skin lesions and is resistant to sodium stibogluconate but successfully treated with liposomal Amphotericin-B.

## Introduction

Kala-azar (Visceral Leishmaniasis) is a deadly disease caused by parasitic protozoa ‘Leishman- Donovan’ (LD body). It lowers immunity and causes persistent fever, anemia, hepatosplenomegaly, and weight loss, and if left untreated, it can be fatal. Post Kala-Azar Dermal Leishmaniasis (PKDL) is a skin disorder that usually develops in 10-20% and about 60% of patients with Visceral Leishmaniasis (VL) with a mean duration of 2-3 years or more and 0-6 months after treatment respectively in the Indian subcontinent and Sudan [1]. However, PKDL can occur in people without a history of visceral leishmaniasis. PKDL patients are healthy except for skin lesions and usually do not feel sick. Sometimes PKDL may coexist with VL. These patients with PKDL and concomitant VL may be more appropriately referred to as Para-kala-azar dermal Leishmaniasis, and they take an intermediate position between VL and PKDL [2]. As far as we know, this is the first case of Para Kala- Azar Dermal Leishmaniasis in Bangladesh. Here we report a sporadic Para kala-azar dermal Leishmaniasis in a chronic hepatitis-B infected patient.

## Case presentation

A 25-year-old male presented to a tertiary care medical college hospital in Dhaka with a left-sided upper abdominal lump and multiple skin lesions all over the body for the last 12 years. An abdominal lump was very slowly increasing in size, painless, causing a mild dragging sensation and the skin lesions were present primarily on his back and abdomen, depigmented, painless, and non-itchy. He denied a current history of fever, loss of appetite, weight loss, cough, jaundice, hematemesis, melaena, and blood transfusion. On query, he gave a history of kala-azar 15 years back and treated with injectable medication but could not mention the name of the drug. He was a known case of a chronic hepatitis-B patient on antiviral (Entecavir 1 mg once daily).

**Figure 1:**
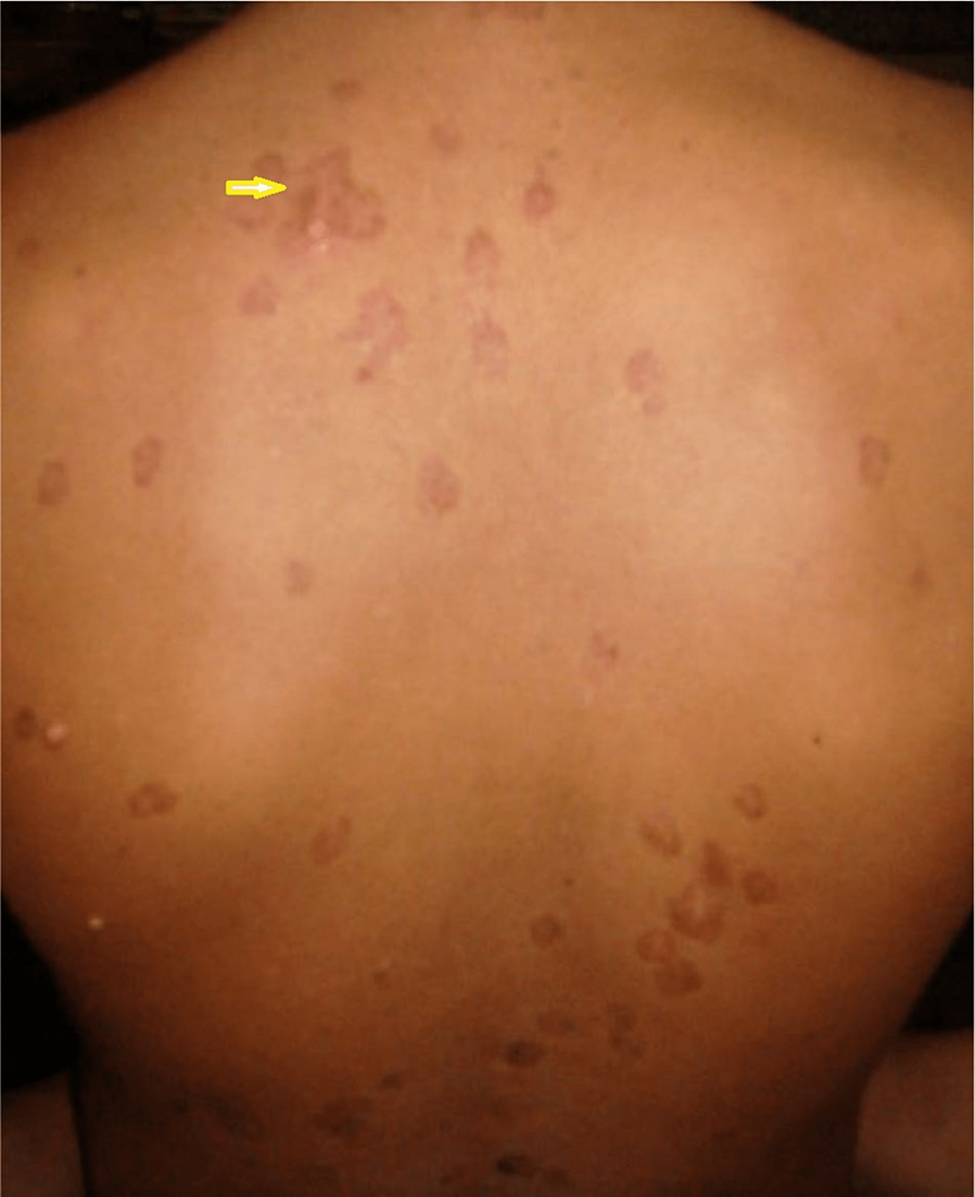
multiple hypo and hyper pigmented macular and maculopapular non-scaly skin lesions present all over the body, mostly on the back and abdomen (yellow arrow)

Examination revealed multiple hypo and hyperpigmented macular and maculopapular non-scaly skin lesions present all over the body, mainly on the back of the chest and abdomen. Lesions were a few millimeters (0.3-0.7 mm) (those hypo-pigmented) to a few centimeters (1-3 cm) (those hyper-pigmented) in diameter (Fig 1), and sensation over the lesions was intact. Other vital parameters were normal. On abdominal examination, there was firm, non-tender hepato-splenomegaly (liver 4 cm from the right costal margin and spleen 6 cm from its long axis).

**Figure 2:**
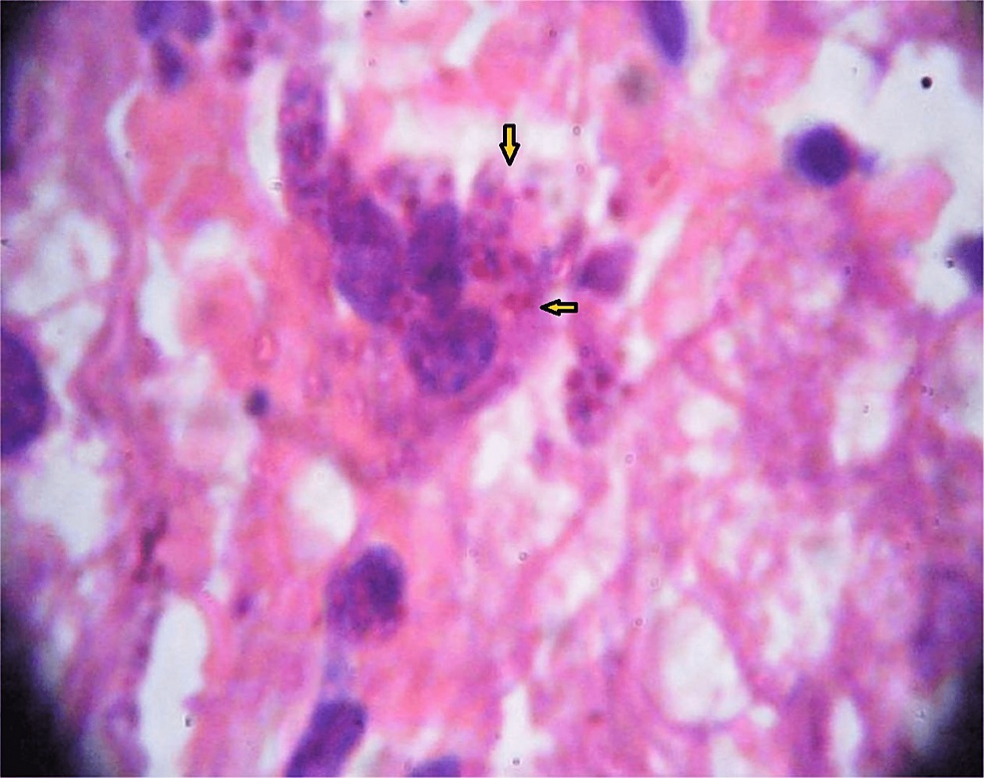
Liver biopsy shows presence of Leismania donovani (LD) bodies in macrophage (yellow arrow)

Slit skin smear from the skin lesions and splenic aspiration showed the presence of LD bodies. A liver biopsy in another tertiary hospital showed the presence of LD bodies without any evidence of liver fibrosis (fig 2). So, we finally diagnosed him with a case of Para Kala-Azar Dermal Leishmaniasis with chronic active Hepatitis-B virus infection. Laboratory information are given in the table 1.

**Table 1:** Investigations profile ESR- Erythrocyte sedimentation rate, GIT- Gastrointestinal tract, HBsAg- Hepatitis B virus surface antigen, HBcAg- Hepatitis B virus core antigen, HCV- hepatitis C virus ICT- Immunochromatographic test, LD- Leishman Donovan

Parameters	Result	Reference
Hemoglobin	10.8 g/dl	11.5-15.5 g/dl
ESR	48 mm in 1^st^ hour ( westergren method)	0-10 mm in 1^st^ hour
White blood cell count	3200 mm^3^	4000-11000 mm^3^
Differential Count (white blood cell count)	Neutrophil-64%, lymphocyte- 32%	
Platelet Count	1,20000 mm^3^	1,50000-4,50000 mm^3^
Urine Routine Examination	Normal	
S. Bilirubin	0.8 mg/dl	0.3-1.2 mg/dl
Alanine aminotransferase	32 U/L	10-40 U/L
S. Albumin	35 g/L	35-50 g/L
Prothrombin Time	14 second	12-16 second
HBsAg	positive	
Anti HBcAg	positive	
HBeAg	positive	
HBV DNA Load	1.1×10^6 ^copies/ml	undetectable
Anti HCV	negative	
ICT for Malaria	negative	
ICT for Kala-azar	positive	
Hemoglobin Electrophoresis	normal	
USG of the Whole Abdomen	Mild hepatomegaly with normal echotexture, Moderate splenomegaly	
Endoscopy Upper GIT	normal	
Splenic aspirate for LD bodies	Present, score 3+	
Liver Biopsy	LD bodies are seen in kuffer cells of the liver, No evidence of parenchymal fibrosis	
Slit skin smear	Amastigotes of Leishman-Donovan with heterogenous morphology, score 2+	

We continued antiviral treatment for hepatitis B. As there is no consensus treatment of para-kala-azar in our national guideline, he was treated as a case of PKDL with an injection of sodium stibogluconate 20 mg/kg deep intramuscular for six cycles (1 cycle = 20 days injection followed by 10 days rest). However, after giving five cycles, we reassessed the case. Skin lesions and spleen size were unchanged after 5 cycles of stibogluconate therapy. So we did a slit skin smear from the lesions and splenic aspiration again, and both showed the presence of an LD body. That is why we considered him resistant to sodium stibogluconate. Then we started injection of Liposomal Amphotericin-B (5 mg/kg^2^ days a week for 3 consecutive weeks) as per national guidelines. After 21 days, we reassessed the case. Skin lesions began to fade and decreased in size gradually, and spleen size decreased. Finally, one month later, a slit skin smear from the lesions and splenic aspiration showed the absence of LD bodies.

## Discussion

Post-kala-azar dermal leishmaniasis (PKDL) is a sequela of visceral leishmaniasis (VL) or kala-azar that develops in 5-10% and 50% of patients in the Indian subcontinent and in Sudan accordingly. It is most prevalent in areas where Leishman-Donovan is the pathogenic parasite. The interval at which PKDL follows VL is 0-6 months in Sudan and 2-3 years in India [2]. PKDL is uncommon in Bangladesh, but in most cases, it remains undetected because it does not cause significant morbidity or mortality and is sometimes confused with leprosy and other skin diseases. The rash of PKDL may be macular, maculopapular, nodular, or plaque-like. Ulceration is uncommon. The lesions initially appear in the perioral region and then spread to the face, trunk, and extremities. Dissemination of the lesions all over the body may mimic lepromatous leprosy [3]. Lymph nodes get enlarged, but most PKDL patients do not have demonstrable parasites in lymph nodes or bone marrow aspiration.

Our patient presented with hypo-hyperpigmented macules and a maculopapular rash all over the body, a common presentation of PKDL. However, unfortunately, in our patient, the skin rash was overlooked. Differentiation from leprosy and other skin diseases is difficult in places where those diseases are endemic. However, PKDL can be diagnosed based on typical characteristics and distribution of skin lesions considering the previous history of Kala-Azar. A slit skin smear examination of the LD body can support a diagnosis. Therefore, physicians must be aware of the differential diagnosis of PKDL.

Usually, immunity after the cure of Kala-Azar is almost lifelong, except for the presence of immunosuppression. Visceral immunity not only protects the host from the recurrence of Kala-Azar by blocking reinvasions of the parasites from the skin in the case of PKDL but also prevents the reactivation of the latent parasites in the viscera [2]. However, a few cases of PKDL associated with Kala-Azar have been reported in the literature. At least seven patients with similar concomitant visceral leishmaniasis and PKDL have been reported from India [4], and another five patients with visceral leishmaniasis and presumably PKDL were seen in Iran [5]. One study from India estimated this to occur in one in every 700 patients with PKDL [6].

Immunosuppression can occur due to intercurrent diseases like measles, malaria, tuberculosis, and HIV infections. it can cause reinvasion of the parasites from the skin to the viscera [7]. Our patient gave no history of such illness. As far as we know, there is not a single case yet reported associated with chronic Hepatitis-B virus infection.

The development of partial immunity after incomplete treatment leads to clinical improvement; however, the parasite can persist. Failure to develop complete immunity may result in the re-establishment of visceral leishmaniasis from the skin [8]. Our patient could not mention the exact duration of previous treatment of visceral leishmaniasis given 15 years ago. So it is more likely that renewed multiplication of the latent parasites in the viscera or re-infection may be the cause of relapse of visceral leishmaniasis in our patient. He came from an endemic area, and the long interval between visceral leishmaniasis and PKDL leaves room for speculation about re-infection.

Still evidence are limited regarding optimal treatment regimen; clinical experience has shown that since 20 mg/kg per day for 30 days was not satisfactory, treatment may need to be prolonged to 2-3 months, or alternative treatment may be necessary. In India, treatment is given with sodium antimony gluconate (SAG) 20 mg/kg per day for 120 days, and the cure rate is 64-92% [9,10]. However, our patient was resistant to sodium stibogluconate and successfully treated with Liposomal amphotericin-B.

## Conclusions

We report this case because it is an uncommon presentation and might be the first case report of concomitant Post Kala-Azar Dermal leishmaniasis (PKDL) with Visceral leishmaniasis (VL) in Bangladesh. Still, we do not know whether any association exists between chronic Hepatitis-B virus infection-induced immunosuppression and reinvasion of the parasites from the skin to the viscera.
